# Impact of an Orthopedic-Delivered ‘One-Stop’ Clinic for Ambulatory Trauma on Emergency Department and Fracture Clinic Pressures During the COVID-19 Pandemic

**DOI:** 10.7759/cureus.15207

**Published:** 2021-05-24

**Authors:** Sophie M Howles, Tariq Mahmood, Sadiah Lala, Andrew Pearse, Charles Docker

**Affiliations:** 1 Trauma and Orthopaedics, Royal Orthopaedic Hospital, Birmingham, GBR; 2 Trauma and Orthopaedics, Worcestershire Acute Hospitals NHS Trust, Worcestershire, GBR

**Keywords:** trauma, fracture clinic, minor injuries, coronavirus, covid-19

## Abstract

Introduction: In March 2020, the World Health Organization declared the severe acute respiratory syndrome (SARS) (coronavirus disease, COVID-19) outbreak as a pandemic. In response to the rising number of coronavirus cases in the United Kingdom, the British Orthopaedic Association (BOA) issued a series of emergency guidelines for the management of trauma and orthopedic patients during the pandemic. In line with this guidance, the orthopedic team at the Worcestershire Royal Hospital set up a ‘one-stop-shop’ minor injuries unit (MIU). This seven-day service provided a direct pathway to the trauma clinic for ambulatory patients who would usually be managed in the emergency department (ED), intending to reduce both the pressure on the ED and the need for further follow-up appointments. The aim of this project was to evaluate the service provided to patients and to inform our practice during the next stages of the pandemic and beyond.

Materials and methods: Data were collected retrospectively from a clinic database, dictated letters, and scanned patient notes. The data collection period was over six weeks from April 6, 2020 to May 18, 2020. Data collected included patient age and gender, time of arrival and departure, grade of reviewing clinician, diagnosis, treatment, and outcome of clinic attendance, including the timing of follow-up.

Results: Some 700 patients were seen in the MIU over six weeks. Some 98% of patients were seen by an orthopedic registrar (resident) or a consultant (attending) and 85% were seen and treated within an hour. Some 71% of patients were discharged after their initial appointment, and only nine patients (1%) required a fracture clinic appointment within 72 hours. A total of 15 patients (2%) re-attended with concerns, and just four of these required additional interventions.

Conclusions: We delivered a seven-day minor injuries service in which the majority of patients received definitive management at first attendance, reducing the demand for fracture clinic appointments. Some 700 patients who would have been treated in the ED were seen in the MIU instead, relieving pressure on the ED. The lessons learned allowed us to plan for the 'second peak' in COVID-19 cases and will inform ongoing practice as we work to recover elective services.

## Introduction

At the start of March 2020, the World Health Organization declared the severe acute respiratory syndrome (SARS) (coronavirus disease, COVID-19) outbreak as a pandemic [1]. This resulted in a significant and rapid restructuring of orthopedic services throughout the United Kingdom, with the cancellation of all elective surgery, and a move towards nonoperative treatment for injuries that are not life- or limb-threatening [2]. In response to the rising number of coronavirus cases in the United Kingdom, the British Orthopaedic Association (BOA) issued a series of emergency guidelines for the management of trauma and orthopedic patients during the coronavirus pandemic. In line with this BOA guidance [3], the orthopedic outpatient team at Worcestershire Royal Hospital, Worcester, United Kingdom set up a ‘one-stop-shop’ minor injuries clinic for the management of ambulatory orthopedic trauma and minor injuries during the first peak of COVID-19. This service was run from 08:00 to 20:00 Monday to Friday and 09:00-17:00 at weekends, providing a direct pathway to trauma and orthopedic clinic for patients who would usually be managed in the ‘minor injuries’ stream within the ED. This included all ambulatory patients with suspected fractures, soft tissue injuries, wounds, and small joint dislocations. A ‘minor injuries pathway’ was created in conjunction with the ED to allow appropriate patients to be sent directly to the MIU by the ED triage nurse.

There were several aims in running this clinic: firstly, by diverting ambulatory trauma patients away from the ED to MIU we hoped to relieve pressure on the ED during the peak of the pandemic. Secondly, by providing patients with senior-led definitive management at the first consultation, we hoped to reduce the need for fracture clinic follow up appointments.

The aim of this project was to evaluate the service that we provided as an MIU, and to audit local outpatient management of patients with traumatic injuries and urgent orthopedic conditions during the COVID-19 pandemic against the guidance set out by the BOA in the Covid-specific ‘BOAST (BOA Standards for Trauma and Orthopaedics)': “Management of patients with traumatic injuries and urgent orthopedic conditions during the coronavirus pandemic” [2]. Through evaluation and discussion of what we have achieved to date, we hoped to draw out learning points that would help to inform the care that we provide to patients during the next stage of the pandemic and beyond, as we begin to rebuild elective services.

## Materials and methods

Data were collected retrospectively from several sources, including a database collected contemporaneously by clinic staff, dictated clinic letters, and scanned patient notes. The data collection period was over six weeks from 06/04/2020 to 18/05/2020. Inclusion criteria were: patients referred to the MIU via the 'minor injuries pathway.' Exclusion criteria were: patients who were not referred to our clinic via the minor injuries pathway, for example, those referred directly from GP to trauma and orthopedics; patients re-attending with the same issue; and those brought back to the clinic by the Trauma and Orthopaedic (T&O) team for follow up. Data collected included patient age and gender, time of arrival and departure, grade of the reviewing clinician, diagnosis, management, and the outcome of clinic attendance, including the timing of follow up. The study follow-up period was at least six weeks for all patients, and details of any complication or re-attendance were recorded, whether the patient re-attended at Worcestershire Royal Hospital, Alexandra Hospital Redditch, or any of the local minor injury units within the Worcestershire Acute Hospitals NHS Trust catchment area.

## Results

Some 700 patients were seen in the MIU over the six-week data collection period. Some 51 patients were excluded as they were not referred via the MIU pathway. Of these excluded patients, 22 were attending for follow up (e.g. post-op wound check), 15 were re-attendances, and seven were referred by an alternative pathway such as direct GP referral to the trauma team, or referral from an ED clinician. Six patients were excluded as there was insufficient documentation available. Of the 649 included patients, 299 were male and 350 female. The youngest patient was six months old and the oldest 96 years old. Some 134 patients were aged under 18 years, 400 patients were 18-65 years, and 115 patients were over 65 years. Patients attended the clinic with a variety of complaints, and for analysis, these were categorized by the documented clinical diagnosis as described in Table 1. The most common diagnoses were soft tissue injuries and fractures, with wounds, lacerations, and foreign bodies making up a significant proportion.

**Table 1:** MIU attendances categorized by diagnosis. MIU, minor injuries unit

Diagnostic category	Number of patients	Proportion of patients (%)
Soft tissue injury	225	35
Closed fracture	177	27
Open fracture	10	2
Dislocation	10	2
Wound/laceration/foreign body	142	22
Infection	23	4
Head Injury (+/- laceration to head or face)	24	4
Chronic Issue (+/- exacerbation)	38	6

**Figure 1:**
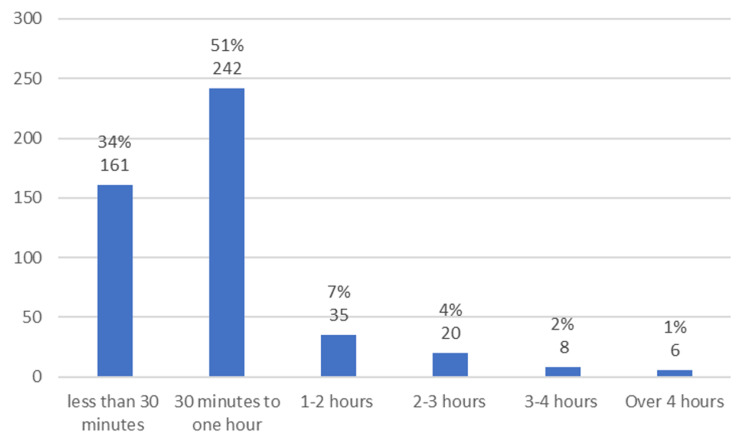
Bar chart showing the time spent in the department

A consultant was present in the MIU each day and 98% of patients were seen by T&O middle grade or consultant. Figure 1 shows the amount of time that patients spent in the department. Some 85% of patients were seen and treated within an hour of arrival. Only six patients (1%) were in the department for over four hours: of these, two patients required inpatient admission, one required referral and transfer to a tertiary center and the final three required procedural intervention under local anesthesia.

**Figure 2:**
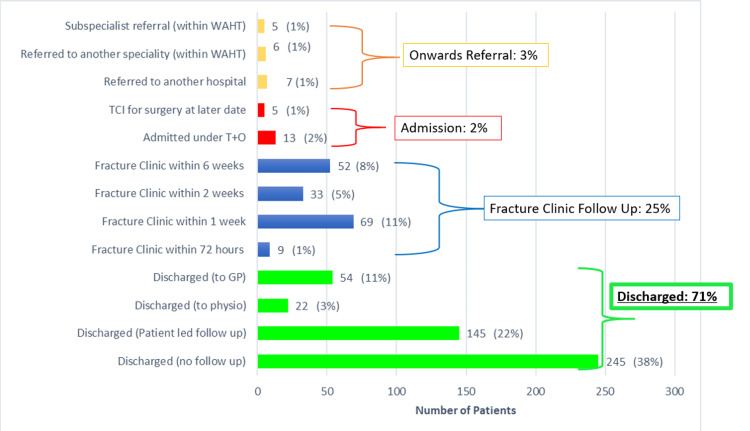
Bar chart showing clinic outcome following MIU consultation, including any follow up planned MIU, minor injuries unit

Some 71% of patients were discharged from their initial appointment, either without follow up, with a patient-instigated follow up, or to primary care or physiotherapy. A total of 25% required fracture clinic follow up with only nine patients (1%) given a fracture clinic appointment within 72 h. All nine of the patients requiring early review had either infections or high-risk wounds. The remaining patients were given a timed fracture clinic appointment at either one, two, or six weeks depending on their clinical needs. Figure 2 shows the outcome and follow up plan for patients following MIU consultation.

A total of 15 patients (2%) either re-attended the clinic or requested a telephone consultation to discuss concerns, with four of these requiring additional intervention and the rest being discharged with further explanation and reassurance.

Of the 187 patients with confirmed or suspected fractures (whether open or closed), 49% of patients were discharged from their initial MIU consultation. Two patients, both of whom had open fractures, were required to follow up in fracture clinic within 72 hours for wound review, five were admitted for surgery (either on the same day or at a later date), and the remaining patients received timed follow up at one, two, or six weeks. Of the patients presenting with acute fracture, 74% were managed with an alternative to full plaster casts. There were no reported complications of this management, with at least a six-week follow up period on each case.

**Figure 3:**
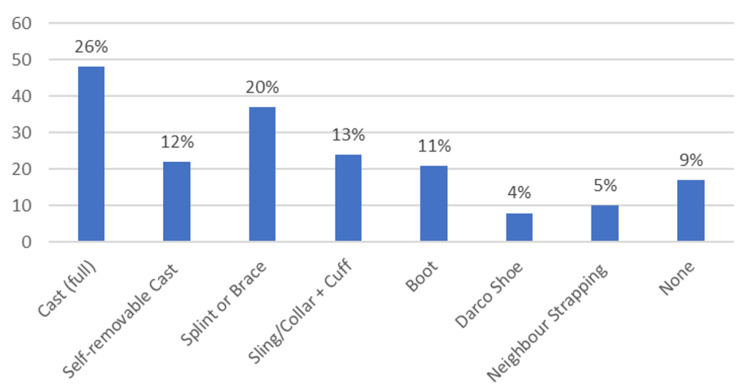
Bar chart showing method of immobilization for patients with confirmed fracture

The bar chart in Figure 3 shows the method of immobilization used for patients with confirmed fracture.

## Discussion

Some 700 patients who would have otherwise been treated in the ED were seen in the minor injuries clinic during the six-week data collection period.

The fact that only nine of the patients seen in our MIU required a fracture clinic appointment within the following 72 h suggests that early orthopedic senior decision-making reduced the need for fracture clinic follow-up. Even within the cohort of patients with fractures or suspected fractures, 49% were discharged from their initial MIU appointment, and the remainder were given appropriately timed follow-up, at one, two, or six weeks. Had those patients been seen in ED, they would have been given a fracture clinic appointment within 72 h of attendance, as per BOAST ‘Fracture Clinic’ guidelines [4]. We acknowledge that there is a lack of control data for this study, however, the unprecedented nature of the first COVID surge meant that this system was implemented rapidly without pre-intervention assessment. Whilst lessons learned during the pandemic may be transferable to routine care, the pandemic provides an unusual set of circumstances, including changes to elective and primary care services, and national lockdown affecting patient behaviors and injury patterns. For this reason, we feel that a direct comparison with pre-pandemic practice is of limited value.

The coronavirus pandemic is likely to affect trauma and orthopedic services for the foreseeable future, and as such, we need to consider clinic footfall carefully whilst social distancing measures remain in place, especially as we work to restore some elective services. Furthermore, this project has created a unique opportunity to appraise a more direct patient pathway and potential improvement in the patients’ experience with a one-stop-shop approach. The results of this study suggest that patients do re-attend if they have concerns, so we should continue to question the need for investigation and additional appointments, with the patient-instigated follow up as a default, unless further review would change the management of their injury.

The results of this study have been presented within our trust to both orthopedic and ED teams. As elective work resumed in Summer 2020, the management of minor injuries was handed back to the ED, and following this, we saw a rise in the number of patients attending fracture clinics. We explored ways in which we could address this, including having a senior trauma and orthopedic decision-maker present in the ED during the peak hours for trauma and minor injuries and implementing a ‘virtual’ fracture clinic.

Unfortunately, December 2020 saw a significant increase in COVID-19 cases in the United Kingdom. As we entered this ‘second peak’, elective orthopedic work once again came to a halt, and pressure on the ED increased. In response to this, the non-ED MIU format returned, with extended hours at weekends based on the patient numbers and peak times identified previously. This has allowed us to provide senior-led minor injuries cover for 12 h a day and 7 days a week with a view to once again relieve pressure on the ED and decrease the need for fracture clinic follow-up.

## Conclusions

Some 700 patients who would have otherwise been treated in the ED were seen in the minor injuries clinic, relieving pressure on the ED. The majority of these received definitive treatment during their first visit, reducing the demand for further fracture clinic appointments. Patient-instigated follow-up was used effectively and alternatives to full casts reduced the need for face-to-face follow-up. The lessons learned have helped to inform the way that we are delivered care in our hospitals as we begin to recover elective services, and enabled us to react quickly and efficiently when COVID case numbers started to rise again in December 2020. Sharing our experiences with our ED colleagues created discussion and shared learning, which will help us to make positive changes in the future.
